# Metatranscriptomics of the Hu sheep rumen microbiome reveals novel cellulases

**DOI:** 10.1186/s13068-019-1498-4

**Published:** 2019-06-20

**Authors:** Bo He, Shuwen Jin, Jiawen Cao, Lan Mi, Jiakun Wang

**Affiliations:** Institute of Dairy Science, College of Animal Sciences, Zhejiang University, Hangzhou, 310058 China

**Keywords:** Rumen microbiome, Metatranscriptomics, Cellulase

## Abstract

**Background:**

Cellulosic biomass has great potential as a renewable biofuel resource. Robust, high-performance enzymes are needed to effectively utilize this valuable resource. In this study, metatranscriptomics was used to explore the carbohydrate-active enzymes (CAZymes), especially glycoside hydrolases (GHs), present in the rumen microbiome of Hu sheep. Select CAZymes were experimentally verified and characterized after cloning and expression in *E. coli*.

**Results:**

The metatranscriptomes of six Hu sheep rumen microbiomes yielded 42.3 Gbp of quality-checked sequence data that represented in total 2,380,783 unigenes after de novo assembling using Trinity and clustered with CD-HIT-EST. Annotation using the CAZy database revealed that 2.65% of the unigenes encoded GHs, which were assigned to 111 different CAZymes families. *Firmicutes* (18.7%) and *Bacteroidetes* (13.8%) were the major phyla to which the unigenes were taxonomically assigned. In total, 14,489 unigenes were annotated to 15 cellulase-containing GH families, with GH3, GH5 and GH9 being the predominant. From these putative cellulase-encoding unigenes, 4225 open reading frames (ORFs) were predicted to contain 2151 potential cellulase catalytic modules. Additionally, 147 ORFs were found to encode proteins that contain carbohydrate-binding modules (CBMs). Heterogeneous expression of 30 candidate cDNAs from the GH5 family in *E. coli* BL21 showed that 17 of the tested proteins had endoglucanase activity, while 7 exhibited exoglucanase activity. Interestingly, two of the GH5 proteins (Cel5A-h28 and Cel5A-h11) showed high specific activity against carboxymethylcellulose (CMC) and *p*-nitrophenyl-β-d-cellobioside (pNPC) (222.2 and 142.8 U/mg), respectively. The optimal pH value for activity of Cel5A-h11 and Cel5A-h28 was 6.0 for both enzymes, and optimal temperatures were 40 and 50 °C, respectively. Both enzymes retained over 70 and 60%, respectively, of their original activities after incubation at 40 °C for 60 min. However, their activities were rapidly diminished upon exposure to higher temperatures. Cel5A-h11 and Cel5A-h28 retained more than 80 and 60% of their maximal enzymatic activities after incubation for 16 h in buffered solutions in the pH range from 4.0 to 9.0.

**Conclusion:**

The metatranscriptomic results revealed that the rumen microbiome of Hu sheep encoded a repertoire of new enzymes capable of cellulose degradation and metatranscriptomics was an effective method to discover novel cellulases for biotechnological applications.

**Electronic supplementary material:**

The online version of this article (10.1186/s13068-019-1498-4) contains supplementary material, which is available to authorized users.

## Background

Cellulose, the major component of plant biomass, is a linear polysaccharide composed of β-1,4-linked d-glucose units. Using various technologies and especially biotechnological processes, cellulosic biomass can be converted to a variety of products, including pulp and paper, textile and animal feed [1, 2]. Moreover, with the rising global energy demands, the abundant and renewable cellulosic biomass represents a possible alternative resource to fossil energy sources [3], and cellulases are essential to biomass conversion to biofuels and other valuable products. It was estimated that 73.9 Tg of dried waste crops in the world could potentially be used to produce 49.1 Gl of bioethanol [4].

In nature, the enzyme-mediated breakdown of cellulose involves different cellulolytic enzyme types, the principal ones being endoglucanases (EC 3.2.1.4), exoglucanases (EC 3.2.1.74 and EC 3.2.1.91) and β-glucosidases (EC 3.2.1.21) [5]. In addition to the cellulases identified from numerous sources using classical microbiological approaches [6, 7], high-throughput omics methods have accelerated cellulase discovery, using techniques that obviate the need for microbial culturing [8–12]. The larger the enzyme repertoire is, the greater the potential to discover new enzymes with robust activities. As herbivores, ruminant animals harbour a highly diverse ecosystem of microorganisms in their gut, especially the rumen, which contains numerous unknown cellulases [13, 14]. Although some researchers have predicted many biomass-degrading gene candidates from the rumen using metagenomics [8, 15], only a few studies have evaluated their expression profiling or experimentally verified the activities of the enzymes encoded by these genes [15, 16]. In China, Hu sheep constitute an important livestock for meat production. This is due to the animal’s excellent prolificacy, rapid growth and ability to adapt to poor-quality feeds and diverse environments [17]. Metatranscriptomic investigation of the cellulolytic system in Hu sheep rumen will enhance understanding of forage digestion, pinpoint the key enzymes and perhaps reveal ones that can be useful for industrial bioconversion processes.

In this study, metatranscriptomics and heterogeneous gene expression were used to explore and evaluate novel cellulase genes from Hu sheep rumen. Our results show that a large number of carbohydrate-active enzymes (CAZymes) are expressed in the rumen of Hu sheep, with *Bacteroidetes* and *Firmicutes* being the major producers of these enzymes. Moreover, novel genes encoding putative cellulases belonging to glycoside hydrolase family 5 (GH5) were identified. Subsequent cloning, heterologous expression in *E. coli* and purification of these confirmed that the gene products are cellulases.

## Results

### Overview of the metatranscriptomes

On average, 49 million raw sequence reads were obtained from the metatranscriptome of each sheep, and a total of 42.3 Gbp of high-quality sequences were obtained after removing the adapters and quality filtering. The Q20 and Q30 base percentages of each sample were above 96.5 and 91.4%, respectively. The average GC content was 47.2% (Additional file 1: Table S1). A total of 2,380,783 unigenes were identified after de novo assembling using Trinity and clustered with CD-HIT-EST. The length of these unigenes ranged from 251 to 40,135 bp, with an average length of 515 bp (Additional file 2: Table S2 and Additional file 3: Figure S1). Approximately 47.3% of the sequences of each metatranscriptome were successfully mapped to the assembled unigenes (Additional file 4: Table S3). Most of the FPKM (fragments per kilobase of transcript sequence per millions of base pairs sequenced) values of the assembled unigenes were below 10 (Additional file 5: Figure S2).

### CAZyme annotation and taxonomic assignment

**Fig. 1:**
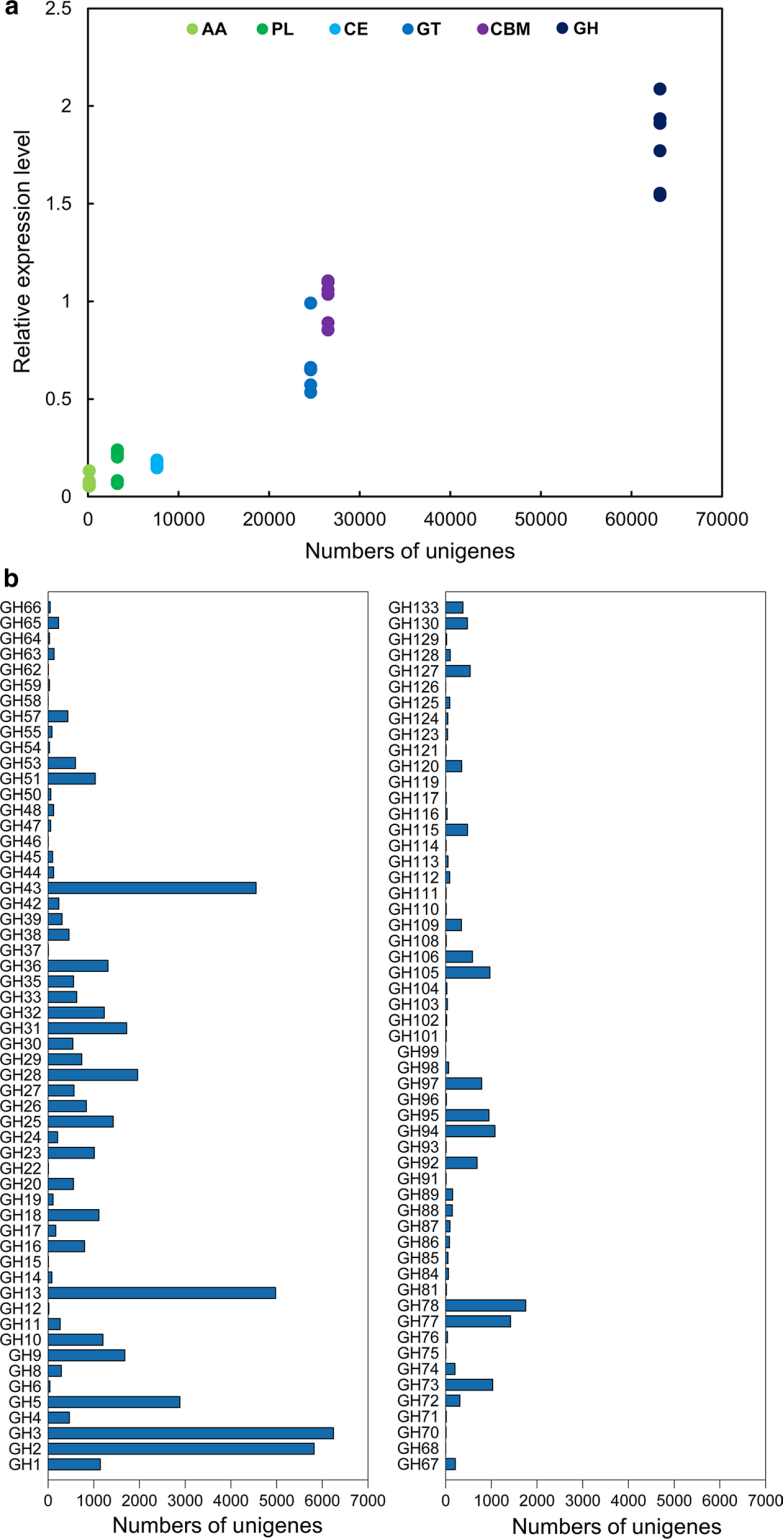
Summary of annotated CAZyme genes. **a** Number and relative level of expression of unigenes annotated to CAZy families. AA, auxiliary activities; CBM, carbohydrate-binding modules; CE, carbohydrate esterases; GH, glucoside hydrolases; GT, glycosyltransferases; PL, polysaccharide lyases. **b** The GH families represented by the unigenes identified in the metatranscriptomes

A total of 2,380,783 unigenes were detected, of which 110,517 were predicted to encode 125,252 putative CAZymes (n.b. each unigene can encode several CAZyme domains and hence the difference between the number of unigenes and the number of putative CAZymes). These predicted CAZymes included 63,153 GHs, 24,599 glycosyltransferases (GTs), 7631 carbohydrate esterases (CEs), 3245 polysaccharide lyases (PLs), 138 auxiliary activities (AAs) and 26,486 CBMs. The GH genes had the highest expression level among all the annotated CAZyme genes (Fig. 1a). The predicated GH genes were annotated to 111 different GH families, with the largest families being GH2, GH3, GH13 and GH43 (Fig. 1b). A total of 14,489 unigenes were annotated to 15 known cellulase-containing GH families (GH1, GH3, GH5, GH6, GH8, GH9, GH12, GH30, GH44, GH45, GH48, GH51, GH74, GH116 and GH124), with GH3, GH5 and GH9 being together represented by 10,810 unigenes (74.6%) (Fig. 1b).

Of the 14,489 putative cellulase genes, only 1.3% showed 95–100% amino acid sequence identity to known proteins deposited in the CAZy database; 14.0% had 75–95% sequence identity; and the rest (84.7%) had less than 75% sequence identity. When compared to the local NCBI nr database using Blastx, 6.4% of the putative genes showed 95–100% amino acid sequence identity; 36.7% had 75–95% sequence identity; and the rest (63.3%) had less than 75% sequence identity (Additional file 6: Table S4).

**Fig. 2:**
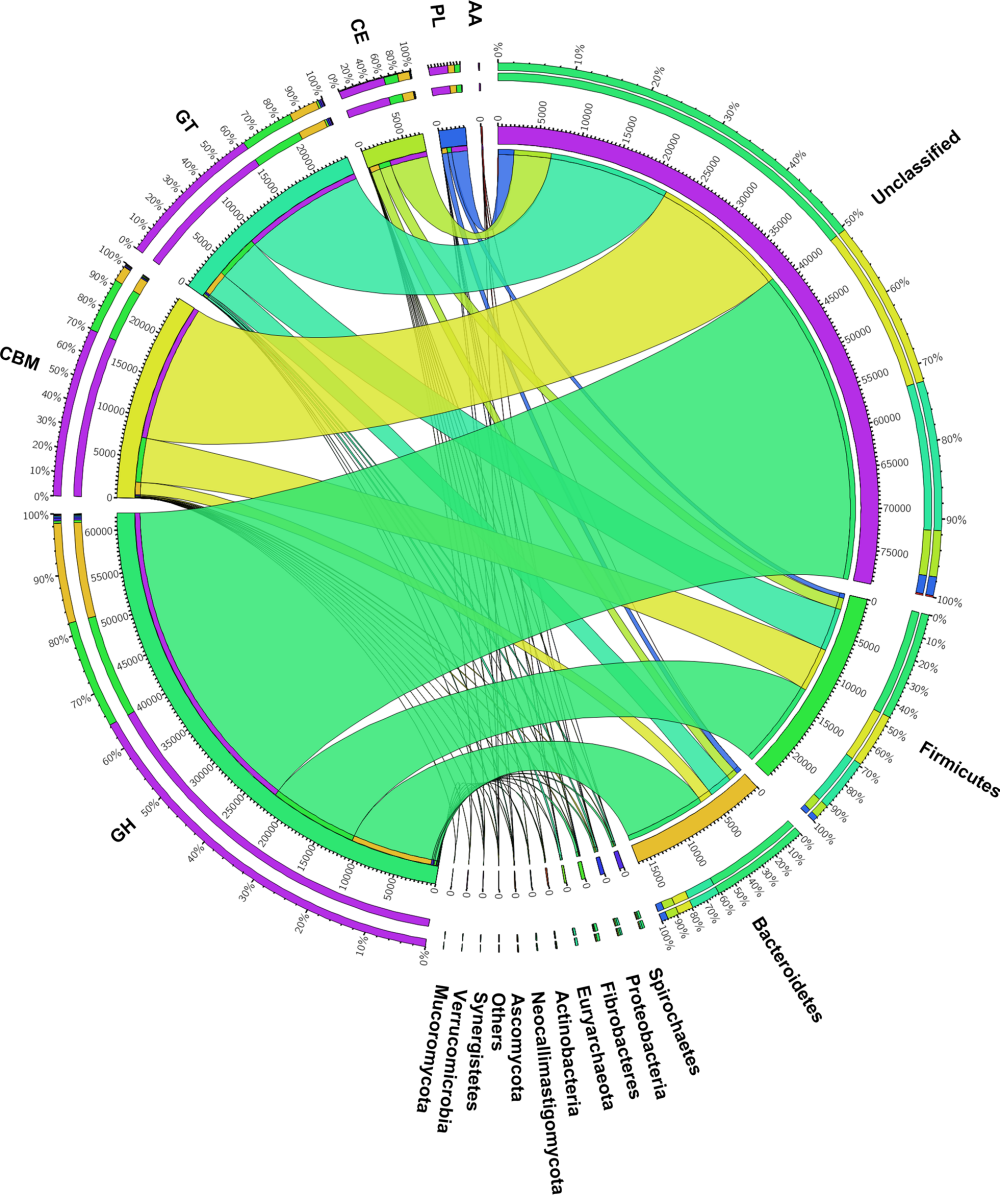
Taxonomic distribution (at phylum level) of the predicted CAZymes identified from the metatranscriptomes. CAZyme families and the corresponding phyla are shown on the left- and right-hand sides, respectively. The outermost ring designates the relative abundance of a given CAZyme family (left) and the relative abundance of unigenes from a given phylum (right); the inner ring designates the total number of unigenes encoding a given CAZyme class (left) and the total number of CAZymes associated with the given phylum. The width of the bars between a given phylum and a given CAZyme family indicates their relative abundance compared to that in the other phyla

Of the total 110,517 unigenes encoding CAZymes, the majority (88.6%) were of bacterial origin, while others were of eukaryotic (1.4%), archaeal (0.3%) or viral (0.04%) origin. However, 10,794 (9.8%) unigenes could not be assigned reliably to any of these groups. *Firmicutes* and *Bacteroidetes* were the largest phyla to which the predicated GHs, GTs, CEs, PLs and CBMs were taxonomically assigned (18.7 and 13.8%, respectively, of total unigenes encoding CAZymes), while *Euryarchaeota* was the major microbial origin of the predicted AAs. Only 1.9% of the CAZymes unigenes were taxonomically assigned to the other 27 phyla (Additional file 7: Table S5), while the remaining CAZymes unigenes (65.6%) could not be assigned to any known phylum of microbes (Fig. 2).

### Cellulase gene identification

Of the 14,489 putative cellulase-encoding unigenes, 4225 were predicted to have a long (≥ 600 bp) ORF (Additional file 8: Table S6). From these ORFs, 2151 catalytic domains (CDs) annotated to 11 GH families and 147 CBMs annotated to 8 CBM families were identified. Similar to the distribution of the unigenes, most of the CD-containing ORFs were annotated to the GH3 (41.6%), GH5 (20.3%) or GH9 (12.4%). CBM_3 and CBM_4_9 were the predominant CBM modules among these ORFs (Table 1).

**Table 1:** Abundance of putative cellulase domains and carbohydrate-binding modules detected in the rumen of Hu sheep

Family^a^	ORF counts^b^	Domain counts^c^	Pfam module^d^	Family ID^e^
GH1	145	151	PF00232	Glyco_hydro_1
GH3	895	1075	PF00933 PF01915	Glyco_hydro_3 Glyco_hydro_3_C
GH5	436	444	PF00150	Cellulase
GH6	4	4	PF01341	Glyco_hydro_6
GH8	54	59	PF01270	Glyco_hydro_8
GH9	266	270	PF00759	Glyco_hydro_9
GH30	68	86	PF02055 PF17189 PF14587	Glyco_hydro_30 Glyco_hydro_30C Glyco_hydr_30_2
GH44	17	18	PF12891	Glyco_hydro_44
GH45	10	11	PF02015	Glyco_hydro_45
GH48	21	27	PF02011	Glyco_hydro_48
GH116	5	7	PF12215 PF04685	Glyco_hydr_116 N DUF608
CBM2	6	6	PF00553	CBM_2
CBM3	44	47	PF00942	CBM_3
CBM4, CBM9^f^	66	67	PF02018	CBM_4_9
CBM6	10	12	PF03422	CBM_6
CBM10	10	23	PF02013	CBM_10
CBM11	6	6	PF03425	CBM_11
CBM35	4	7	PF16990	CBM_35
CBM48	1	1	PF02922	CBM_48

^a^The GHs (glycoside hydrolases) family and CBMs (carbohydrate-binding module) family are classified according to the CAZy database. GH families without a Pfam model are not shown

^b^Number of candidate ORFs (open reading frames) containing at least one domain for a particular GHs or CBMs family

^c^Number of CDs (catalytic domains) or CBMs modules for a particular family

^d^Pfam model associated with the respective GHs or CBMs family

^e^Pfam name of the catalytic domain associated with the respective CBMs family

^f^CBMs families that cannot be distinguished by Pfam model were combined

### Phylogeny and microbial origin of the cellulase candidates annotated to the GH5 family

**Fig. 3:**
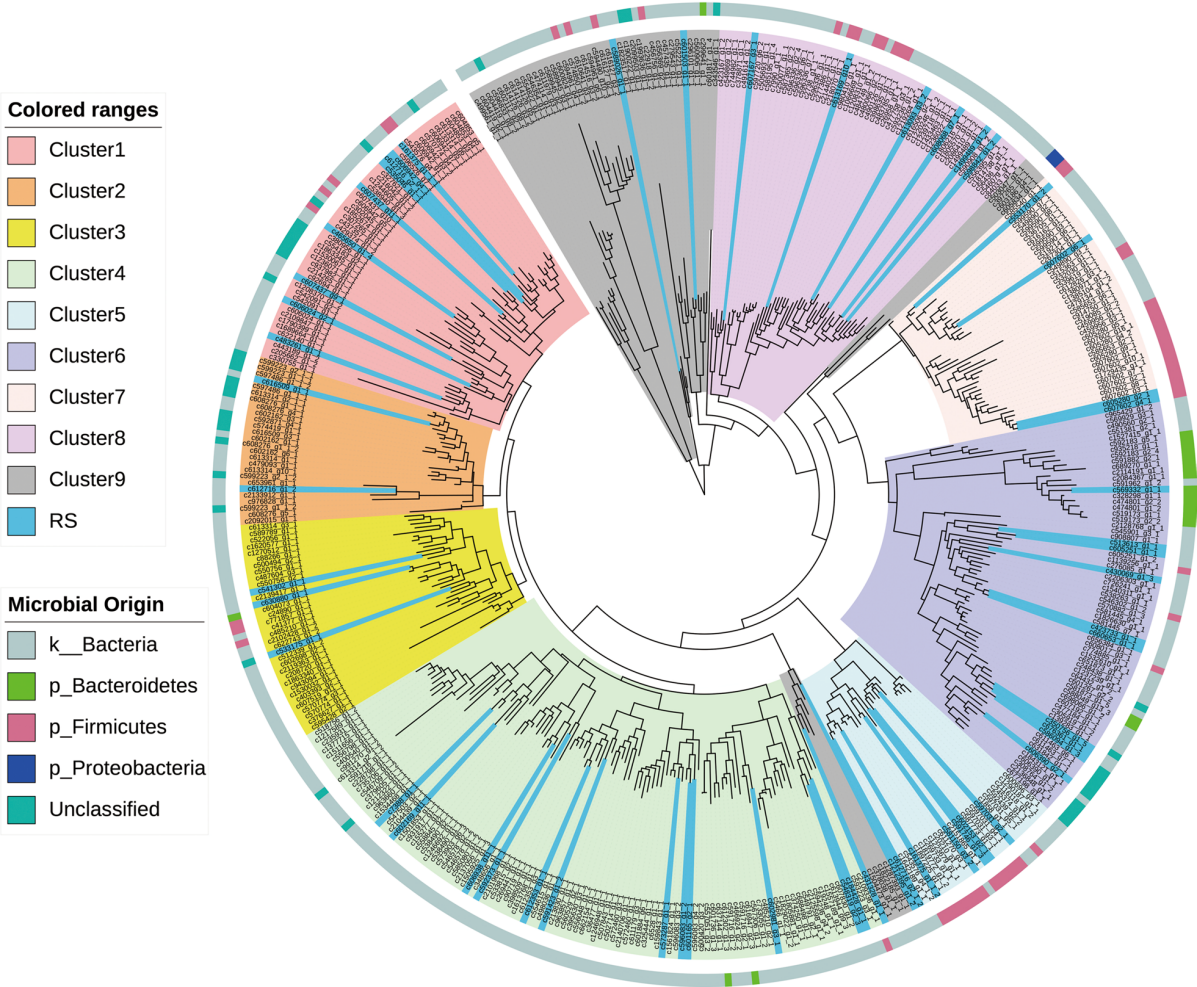
Phylogeny and microbial origin of the catalytic domains of cellulase candidates assigned to the GH5 family. The clusters were formed according to the evolution distance and labelled with different colour. ‘RS’ represents the genes selected for further validation. The microbial origin of the putative cellulases was drawn as a bar chart (the outermost ring) according to the programming file provided by the website

Phylogenetic analysis showed that most of the GH5 cellulase candidates grouped into eight unique clusters (Clusters 1 to 8 in Fig. 3), while a few putative cellulases failed to cluster (Cluster 9 in Fig. 3). The majority of putative cellulases were of bacterial origin (Fig. 3). The FPKM value, which indicates the expression level, of most the cellulase genes was below 1, except the genes from cluster 3 that showed relatively high expression levels, with 13 of the 26 members of this cluster having an FPKM value greater than 10 (Additional file 9: Table S7).

### Function validation of selected GH5 genes

Fifty-four ORFs annotated to the GH5 family were selected from all the eight phylogenetic clusters and the non-clustered segment (labelled as ‘RS’ in Fig. 3) to experimentally verify whether they do encode active cellulases. Using respective specific primers for each of the ORFs and RT-PCR, 30 of the 54 (55.6%) ORFs yielded the PCR products with the predicted size. After sequencing, 21 of the 30 PCR products had 100% sequence identity to the predicted candidate genes, and the sequence identity of the other 9 PCR products was higher than 95%. These 30 PCR products shared 38 to 99% amino acid sequence identity with known proteins, with most of them having an amino acid sequence identity less than 67%. The protein sequences were between 303 and 807 amino acids in length (Table 2).

**Table 2:** Properties of the 30 cellulase candidates from the GH5 family used to determine their cellulolytic activity

ORFs^a^ ID	Sample ID	Amino acid length	Best BLAST hit vs. NCBI (enzyme annotation)	Accession number	Identity %	Signal peptide^b^
c589026_g1_1	Cel5A-h1	344	Glycoside hydrolase	WP_022932641.1	70	N
c607167_g3_1	Cel5A-h2	556	Glycoside hydrolase	WP_020965515.1	49	Y
c350756_g1_5	Cel5A-h10	744	Glycoside hydrolase family 5 protein	WP_097035831.1	66	Y
c1499489_g1_2	Cel5A-h11	317	Glycoside hydrolase family 5 protein	WP_031559633.1	68	N
c676361_g1_1	Cel5A-h12	721	T9SS C-terminal target domain-containing protein	WP_101480019.1	52	Y
c423733_g1_1	Cel5A-h13	303	Glycoside hydrolase family 5 protein	WP_013498507.1	99	N
c7388_g1_1	Cel5A-h14	499	Endoglucanase	WP_115153074.1	57	N
c430069_g1_3	Cel5A-h15	512	Endoglucanase	CDC18601.1	52	N
c592373_g1_1	Cel5A-h16	633	Glycoside hydrolase family 5 protein	WP_084156318.1	55	Y
c581150_g3_2	Cel5A-h17	602	Putative carbohydrate-active enzyme	ADX05709.1	64	N
c602169_g11_1	Cel5A-h21	437	Cellulase	ADU86908.1	68	N
c535046_g1_1	Cel5A-h23	410	Glycoside hydrolase family 5 protein	WP_013499238.1	99	Y
c613169_g10_1	Cel5A-h24	335	Endoglucanase	WP_103869183.1	65	N
c660653_g1_1	Cel5A-h26	304	Hypothetical protein RASY3_16565	EXM37921.1	95	N
c602981_g3_1	Cel5A-h27	698	Glycoside hydrolase family 5 protein	WP_072812340.1	92	Y
c630880_g1_1	Cel5A-h28	480	Endoglucanase	CBL34359.1	50	Y
c533175_g1_1	Cel5A-h31	415	Cellulase	AHF24954.1	87	Y
c591423_g1_2	Cel5A-h32	609	Multispecies: hypothetical protein	WP_072418845.1	44	Y
c569332_g1_1	Cel5A-h35	617	Multispecies: glycosyl hydrolase family 5	WP_031534432.1	48	Y
c606942_g4_1	Cel5A-h37	731	Endoglucanase	WP_028517111.1	48	Y
c608068_g7_1	Cel5A-h38	343	Hypothetical protein	WP_044916478.1	68	N
c597031_g2_1	Cel5A-h41	445	Glycoside hydrolase family 5 protein	OUM69972.1	55	N
c573287_g1_1	Cel5A-h42	807	Endoglucanase	CDE12421.1	45	Y
c605251_g1_1	Cel5A-h44	387	Glycoside hydrolase family 5 protein	WP_014775421.1	38	N
c607602_g6_1	Cel5A-h45	593	Cellulose 1,4-beta-cellobiosidase	WP_037304596.1	99	N
c483261_g1_1	Cel5A-h47	365	Glycoside hydrolase family 5 protein	WP_080550482.1	72	N
c607437_g11_1	Cel5A-h49	577	Glycosyl hydrolase family 5	WP_013497304.1	99	N
c607437_g9_1	Cel5A-h50	690	Glycoside hydrolase family 5	WP_100068403.1	83	Y
c586094_g1_3	Cel5A-h53	736	T9SS C-terminal target domain-containing protein	WP_101480019.1	60	Y
c541302_g1_1	Cel5A-h54	433	Glycoside hydrolase family 5 protein	WP_083379902.1	65	Y

^a^ORFs, open reading frames

^b^Presence (Y) of a signal peptide was predicted using the SignalP program

**Fig. 4:**
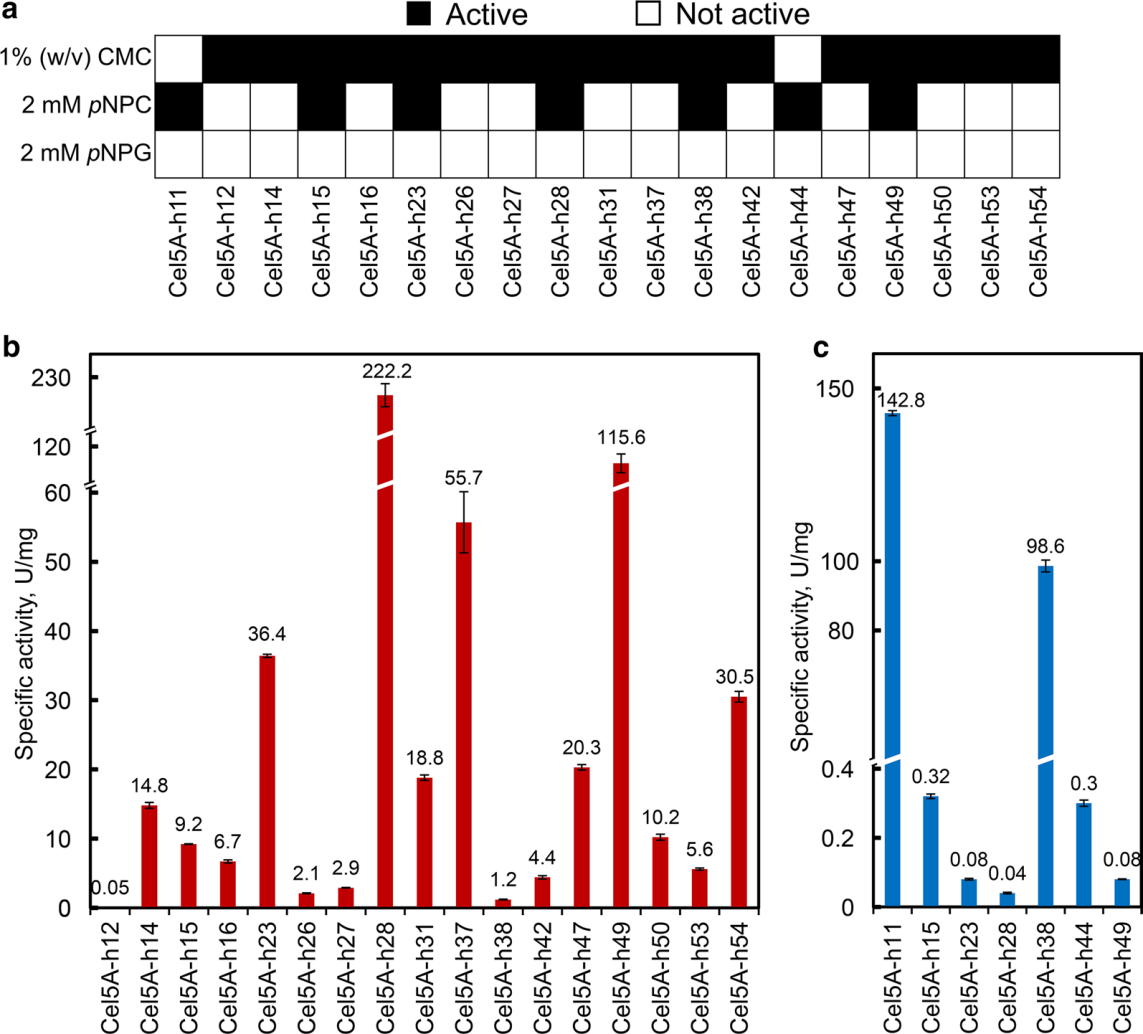
Cellulolytic potential of candidate cellulases on different substrates and the specific activities of the cellulases. **a** Summary of activity assays of 19 cellulase candidates on at least one of the three substrates. **b** The specific CMCase activities of the purified cellulases. **c** The specific activities of the purified cellulases on *p*-nitrophenyl-β-d-cellobioside (pNPC) as substrates

The RT-PCR products of the above 30 ORFs were each cloned and expressed in *E. coli*. Nineteen (63.3%) of the expressed enzymes showed apparent enzymatic activity against at least one of the following substrates: carboxymethyl cellulose (CMC), *p*-nitrophenyl-β-d-cellobioside (pNPC) and *p*-nitrophenyl-β-d-glucopyranoside (pNPG), and 17 exhibited endoglucanase activity, 7 exoglucanase activity and 5 both endo- and exoglucanase activities. No β-glucosidase activity was detected (Fig. 4a). Two of the clones, Cel5A-h28 and Cel5A-h49, had the highest specific endoglucanase activity, 222.2 and 115.6 U/mg, respectively (Fig. 4b), while the other two clones, Cel5A-h11 and Cel5A-h38, had the highest specific exoglucanase activity, 142.8 and 98.6 U/mg, respectively (Fig. 4c).

**Fig. 5:**
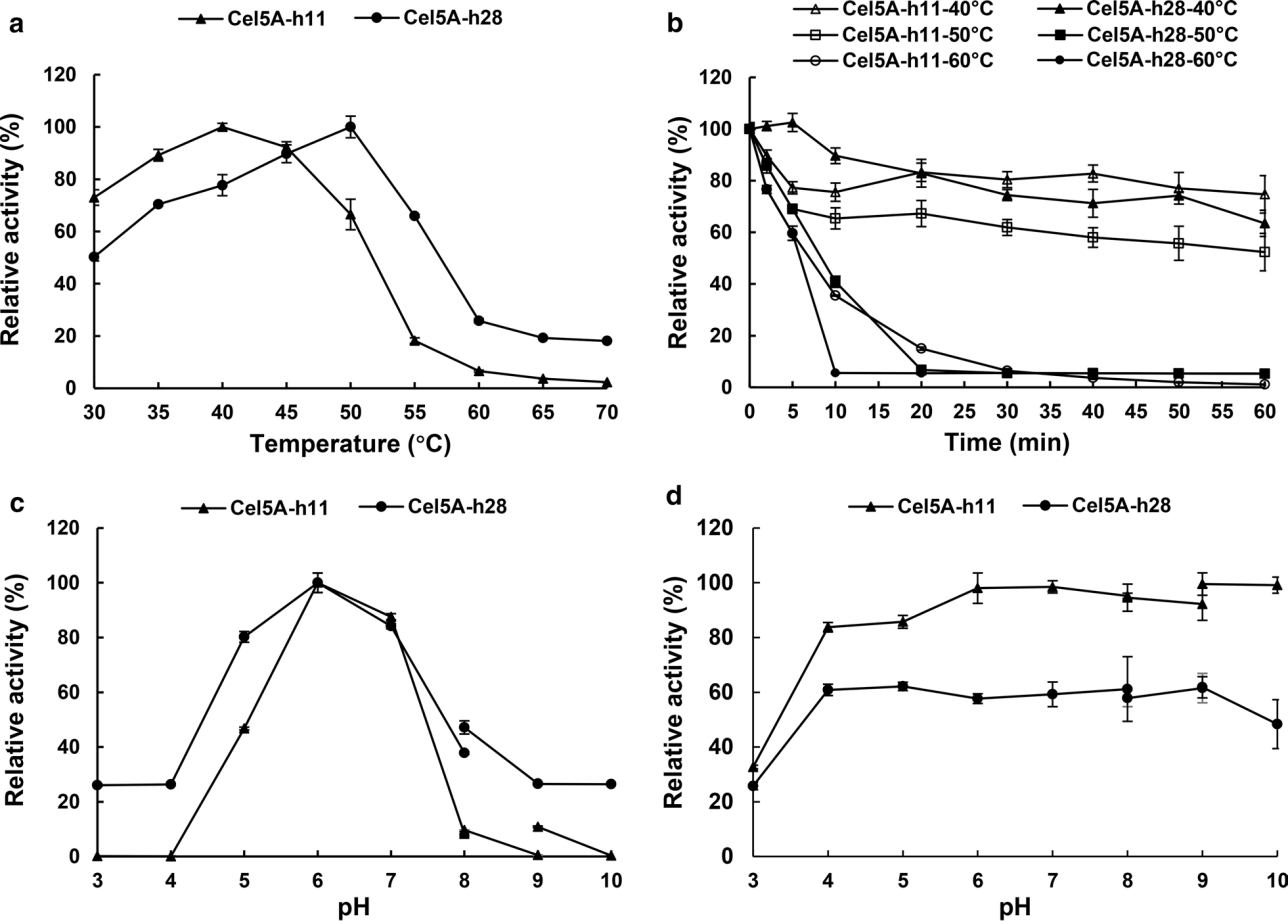
Effect of temperature and pH on the activity of purified recombinant Cel5A-h11 and Cel5A-h28. **a** Temperature effect when tested at the optimal pH 6.0. **b** Residual enzymatic activity at optimal pH and temperature after incubation at different temperatures (40 °C, 50 °C and 60 °C) for different duration (2, 5, 10, 20, 30, 40, 50 and 60 min). **c** pH effect when tested at the optimal temperature of each enzyme (40 °C for Cel5A-h11 and 50 °C for Cel5A-h28). **d** Residual enzymatic activity at optimal pH and temperature after 16 h incubation at 4 °C in different pH range of 3.0–10.0

The temperature optima for Cel5A-h11 and Cel5A-h28 were determined to be 40 and 50 °C (Fig. 5a) when pNPC and CMC were used as the substrates, respectively. Cel5A-h11 and Cel5A-h28 retained over 70 and 60%, respectively, of their original enzymatic activities after incubation at 40 °C for 60 min. However, the enzyme activities of Cela5A-h11 and Cel5A-h28 decreased rapidly when exposed to 50 and 60 °C, respectively (Fig. 5b). The optimal pH for Cel5A-h11 and Cel5A-h28 was both 6.0 (Fig. 5c). Cel5A-h11 and Cel5A-h28 retained more than 80 and 60% of their maximal enzymatic activities after incubation for 16 h in buffered solutions in the pH range from 4.0 to 9.0, respectively (Fig. 5d).

## Discussion

Enzymatic deconstruction of cellulosic biomass to sugars (mainly glucose and cellobiose) depends on the synergistic actions of endoglucanases, exoglucanases and β-glucosidases. Although *Trichoderma reesei* is the most widely used organism to produce commercial enzymes for cellulose hydrolysis, many studies have found natural enzymes from other organisms with better performances [18, 19]. As the enabler of plant fibre degradation in ruminants, the rumen microbiome is enriched with cellulolytic microbes and CAZymes. Several studies have used metagenomics and metatranscriptomics to explore the lignocellulase systems in the rumen of cows and buffaloes [15, 20]. However, this is the first attempt to explore the genetic diversity, expression levels and microbial origin of CAZymes genes in the rumen of Hu sheep using a metatranscriptomic approach. As revealed by the metatranscriptomic results, the Hu sheep rumen microbiome is a rich source of new CAZymes, which can be useful in the conversion of cellulosic biomass including conversion to biofuels.

CAZymes are involved in many biological processes such as carbohydrate metabolism, protein glycosylation and plant biomass synthesis and degradation in different ecosystems [21]. Of the five functional categories of CAZymes, GHs and PLs are involved in glycosidic bond cleavage between two sugar units or between a sugar and a non-sugar moiety [22]. The large repertoire of GHs revealed in the rumen microbiome of the Hu sheep indicates the strong fibre degradation function in the rumen of Hu sheep. It should be noted that AAs, which are a group of ligninolytic enzymes or polysaccharide lytic mono-oxygenases and act in conjunction with the other four categories of CAZymes [23], were expressed at low levels. This is consistent with the need for oxygen in lignin breakdown and the limited lignin degradation in the rumen where oxygen is rare [24].

Cellulases, including endoglucanases, exoglucanases and β-glucosidases, were classified into various GH families, and almost all major cellulase-containing GH families were detected in the rumen microbiome of Hu sheep. The cellulases belonging to GH3, GH5 and GH9 were particularly abundant, while the hemicellulases belonging to GH43 were also enriched. These results indicated the importance of these GH enzymes to the degradation of carbohydrate components of plant cell wall materials in the rumen of Hu sheep. However, most of these predicted CAZymes shared low sequence similarity to known proteins deposited in the CAZy and NCBI nr databases, suggesting that the rumen of Hu sheep harbours novel and uncharacterized cellulases. Consistent with a previous study on the ruminal fibre-associated bacterial community of Holstein cows [25], a considerable proportion (65.6%) of the unigenes encoding CAZyme candidates remained unclassified even at the phylum level, which might be attributed to the relatively short gene sequences or suggest the existence of novel and uncharacterized cellulolytic microbes in the rumen of Hu sheep. Most of the cellulases identified in the present study were taxonomically assigned to *Firmicutes* and *Bacteroidetes*. This is consistent with the fact that these two phyla account for the majority of known cellulolytic bacterial species [26]. Intriguingly, some of the AAs detected in the rumen of Hu sheep were assigned to *Euryarchaeota*. Future research is needed to determine whether these AAs are encoded in the genomes of *Euryarchaeota*, and if so to what extent *Euryarchaeota* contribute to or affect the degradation plant cell materials in the rumen.

To discover full-length cellulolytic genes from our metatranscriptomes of the Hu sheep rumen microbiome, all the putative cellulase-encoding unigenes assigned to 15 GH families were subjected to ORF prediction. The conventional enzyme discovery methods based on overall sequence homology have limited ability to predict enzymes from sequences that have little sequence similarity with known CAZymes [15]. However, similar enzymes often share a few short motifs around the active site, even when the overall sequences are very different [27]. Therefore, the amino acid sequences of the predicted ORFs were searched for catalytic and carbohydrate-binding domains to avoid missing potential novel enzymes. The abundance profile of the CDs in these 15 GH families further confirmed the prediction accuracy and the diversity of putative cellulases in the rumen microbiome of Hu sheep. CBMs enable CAZymes to bind to their substrates [6]. Among all the CBMs families identified in this study, CBM3 accounted for 27.8% of all the predicted CBMs. Because of its ability to bind to crystalline cellulose [28], CBM3 probably plays an important role in the digestion of the crystalline cellulose of plant cell wall biomass.

The GH5 family is a small GH family within the CAZy database, but it contains all the three types of cellulase: endoglucanases, exoglucanases and β-glucosidases. All of the GH5 family gene candidates identified within the metatranscriptomes described in this study displayed a putative ‘cellulase-like’ CD (i.e. a catalytic domain that displays some sort of cellulase activity). Therefore, these were selected for screening, with aim to confirm that were indeed cellulase. A total of 55.6% of the 54 full-length ORFs of the candidate GH5 cellulases were successfully amplified, using RT-PCR and starting from total RNA extract from Hu sheep rumen samples. The amplified ORFs displayed between 38 and 99% amino acid sequence identity with known proteins archived in the NCBI nr database, indicating that most of the predicted genes obtained through de novo assembly of the metatranscriptomic data represent actual genes present in the rumen microbiome of the Hu sheep. In addition, 73.3% of the assembled genes were less than 75% identical to any known protein-encoding genes deposited in the NCBI nr database. These results again indicate that most of the predicted genes represent potentially new CAZymes involved in the cellulose degradation in the rumen of Hu sheep.

The advancement and broad application of high-throughput sequencing technologies have led to a quantitative explosion of putative gene sequences. As of May 6, 2019, a total of 1,246,474 CAZymes (GHs, GTs, PLs, CEs, AAs) genes were deposited in the CAZy database, of which only 10,302 (0.83%) enzymes were biochemically characterized [29]. It is becoming increasingly important to transform these gene sequences into valid information [27]. Cloning and expression of genes provide an opportunity to better understand the functions and diversity of enzymes and promote their biotechnological applications [30]. In the study, 19 of the 30 tested cellulase candidates that were expressed in *E. coli* BL21 displayed enzymatic activity on at least one type of the substrates used in the assays, and several endoglucanases and exoglucanases with high specific activity were obtained compared to some previous studies [31–35]. These results suggest that the candidate genes predicted by our metatranscriptomic strategy are highly enriched with cellulase genes. The biochemical characterization of the recombinant proteins with highest activity of endo- and exoglucanases (Cel5A-h28 and Cel5A-h11) was also assayed. The two enzymes were moderately thermally stable and displayed a stable activity over a broad pH range from 4.0 to 9.0. Inactivity of the remaining cellulase candidates might be due to several reasons, including false-positive prediction of functional domains, inability to express and misfolding of the recombinant proteins in *E. coli* and suboptimal enzymatic assay conditions [15]. It is noteworthy that although five of the expressed proteins showed activity against both CMC and pNPC, the results showed that they only had high activity against one of the substrates, which indicates that simultaneous degradation of cellulose to glucose and/or cellobiose by a single natural enzyme is inefficient. The overall low identification of β-glucosidases might be due to their underrepresentation in the GH5 family in the CAZy database. Therefore, the other putative cellulases of our metatranscriptomic data should be further validated in future studies.

## Conclusion

Metatranscriptomic analysis coupled to heterogeneous expression in *E. coli* enabled us to accurately discover numerous CAZymes, many of which represent new members in the CAZy classification. Microbiomes studied thus far, *Firmicutes* and *Bacteroidetes*, were the major sources of CAZymes and probably the primary degraders of plant biomass in the rumen. The rumen of Hu sheep is a rich source of novel cellulases that can be developed into robust catalytic agents to potentiate the production of biofuels from cellulosic biomass or serve as feed enzymes.

## Methods

### Rumen content sample collection

Six 1.5-year-old healthy cannulated male Hu sheep (63.91 ± 6.18 kg) were used in this study. The sheep were fed only chopped alfalfa ad libitum for 21 days and had free access to fresh drinking water before rumen sample collection. The rumen content was collected from each sheep and immediately snap-frozen in liquid nitrogen and stored at − 80 °C until total RNA isolation.

### RNA isolation and metatranscriptomic sequencing

The total RNA was extracted from each rumen content sample using an RNApure Total RNA Kit (Aidlab, Beijing, China) according to the manufacturer’s instructions and subjected to DNase I (Tiangen, Beijing, China) digestion to remove contaminating DNA. The quality of the RNA and removal of potential contaminating DNA were visually assessed using agarose (1%) gels electrophoresis. Then, the RNA concentrations were confirmed using a NanoPhotometer^®^ spectrophotometer (IMPLEN, CA, USA), and the RNA was quantified using a Qubit^®^ RNA Assay Kit with a Qubit^®^ 2.0 Fluorometer (Life Technologies, CA, USA). The RNA integrity was evaluated using the RNA Nano 6000 Assay Kit and the Bioanalyzer 2100 system (Agilent Technologies, CA, USA).

A total of 3 µg of total RNA per sample was processed for rRNA depletion using a Ribo-Zero™ Gold rRNA Removal Kit (Epidemiology), which combines the Ribo-Zero™ Gold (Human/Mouse/Rat) and Ribo-Zero™ (Bacteria) rRNA removal reagents (Epicentre, Madison, USA). Then, one sequencing library was generated for each sample using the NEBNext^®^ Ultra™ RNA Library Prep Kit for Illumina^®^ (NEB, USA) following the manufacturer’s recommendations. Library quality was assessed on an Agilent Bioanalyzer 2100 system (Agilent, California, USA). The individual libraries were preparated and sequenced on an Illumina HiSeq 4000 platform using the 2 × 150 bp chemistry following the standard protocols provided by the manufacturer (Illumina, San Diego, USA).

### Metatranscriptome data analysis

The raw sequence reads were subjected to filtering of host reads, adapter sequences or poly-N and low-quality (Q < 20) sequences. The Q20, Q30 and GC content of the quality-filtered data were calculated. Ribosomal RNA sequences were removed by comparison with the NCBI rRNA, tRNA and SILVA databases. The remaining quality-filtered sequence reads were assembled de novo into transcripts using Trinity [36] (v.2014-04-13p1) with the default parameters. Then, the transcripts of all the six samples were combined and clustered into unique classes with CD-HIT-EST [37] at 95% identity. After the assembly and clustering of transcripts, the longest sequence of each class was treated as unigene. The expression of each unigene was evaluated as FPKM. The quality of the assembled sequences was assessed by mapping the quality-filtered paired-end reads to the assembled sequences using Bowtie (v2.2.3) with the default parameters implemented in RSEM (RNA-Seq by Expectation Maximization) [38]. The number of reads mapped to each unigene was counted, and the FPKM of each gene was calculated based on the length of the gene and the read counts mapped to that gene [39]. The raw sequence reads were submitted to the NCBI Sequence Reads Archive (SRA) as BioProject PRJNA517369. The accession numbers of the read data were SRR8580849, SRR8580848, SRR8580851, SRR8580850, SRR8580847 and SRR8580846.

### Taxonomic annotation of unigenes

A Blast search (*e*-value < *e*^−5^) of the unigenes was performed against the NCBI nr database. As each sequence may have multiple hits, the taxonomy was estimated with a custom version of the lowest common ancestor (LCA) algorithm implemented in MEGAN in order to ensure its biological relevance [40]. The default parameters were used with the exception that only the hits exceeding a bit score of 50 and a length of more than 25 nucleotides were considered.

### Functional annotation of CAZymes

The CAZy database (version as of 20 October 2014) was used in annotating the unigenes. We firstly obtained the accession number of the CAZymes deposited in the CAZy database and then downloaded all the corresponding sequences from the NCBI nr database based on the accession numbers. A local Blast search was then used to annotate the unigenes by comparing the gene against the downloaded NCBI sequences using an *e*-value ≤ 1*e*−5. Because there might be more than one hits for each unigene, we calculated the Blast coverage ratio (BCR) for both the query and the reference sequences using the following equations

$${\text{BCR}}\_{\text{Ref}} = \left( {{{{\text{Align}}\_{\text{length}}} \mathord{\left/ {\vphantom {{{\text{Align}}\_{\text{length}}} {{\text{Ref}}\;{\text{length}}}}} \right. \kern-0pt} {{\text{Ref}}\;{\text{length}}}}} \right) \times 100\%$$

$${\text{BCR}}\_{\text{Que}} = \left( {{{{\text{Align}}\_{\text{length}}} \mathord{\left/ {\vphantom {{{\text{Align}}\_{\text{length}}} {{\text{Que}}\;{\text{length}}}}} \right. \kern-0pt} {{\text{Que}}\;{\text{length}}}}} \right) \times 100\%$$

where Ref refers to reference sequence and Que refers to query sequence.

The unigenes with a BCR_Ref or BCR_Que < 40% were excluded from further analysis. Also, only the unigenes with a high-scoring segment pairs (HSP) > 60 bits were selected for subsequence analysis in order to ensure biological significance. The predicted CAZymes unigenes were further compared against the NCBI nr database (formatted in May, 2019) using the local Blastx program (*e*-value ≤ 1*e*−5).

### Cellulase ORF prediction and domain prediction

In the CAZy database, one family rarely coincides with one single types of substrate, and thus, many of these families contain enzymes that have different types of CAZymes and EC numbers [41]. The unigenes belonging to 15 GH families (GH1, GH3, GH5, GH6, GH7, GH8, GH9, GH12, GH30, GH44, GH45, GH48, GH51, GH74, GH116, GH124 and GH131) that contain at least one type of cellulase were further analysed, while the GH families that contained few characterized cellulases or had no match with the genes predicted in this study were not further analysed. The unigenes assigned to the above 15 GH families were subjected to six-frame ORF prediction with a minimal length of 600 bp using the getorf algorithm of EMBOSS [42]. The standard code or the code of bacterial was used in accordance with the microbial groups to which GHs had been taxonomically assigned. The amino acid sequences of all the predicted ORFs were subsequently subjected to screen for CDs of GHs or for CBMs using HMMER hmmsearch implemented in the Pfam database [43] (Pfam version 31.0 and HmmerWeb version 2.25.0). The cut-off was set to the Gathering Threshold (HMMER) defined in the database [9]. To identify the enzymes most closely related to the ORFs, the corresponding amino acid sequences of the ORFs that each contained one or more CDs were searched against the NCBI nr database for the best hits of known proteins using Blastp. The signal peptide of the proteins was predicted using the SignalP 4.1 server [44]. Because all the predicted ORFs assigned to the GH5 family were predicted to contain a CD of ‘cellulase-like’, the genes from this family were further analysed for cellulase discovery.

### Phylogeny of the predicted cellulases

The amino acid sequences of the ORFs assigned to the GH5 family were combined into one data matrix (Additional file 10) for phylogenetic analyses using MEGA6. The regions that aligned to the Pfam HMMs were aligned using ClustalW. Phylogenetic trees were generated using the maximum likelihood method with the Jones–Taylor–Thornton model applied and using a bootstrap replication of 500 using MEGA6. The trees were then imported into the online software iTOL [45] for further optimization.

### Verification of de novo assembly

To validate the accuracy of the ORFs predicted from the metatranscriptomic sequence data and assess their expression, full-length ORFs were selected randomly from each of the nine phylogenetic clusters of the GH5 family for experimentally verification. Briefly, one pair of specific primers were designed to amplify the cDNA corresponding to each respective ORF. All the primer pairs were designed to have similar T°m so that all the ORFs could be RT-PCR amplified in the same run. To facilitate cloning into pET-28a, each primer had a 5′ extension of 22-bp sequence homologous to the pET-28a (+) vector (Invitrogen, Shanghai, China) insertion site (Forward: GGCCATGGCTGATATCGGATCC; and Reverse: CTTGTCGACGGAGCTCGAATTC) (Additional file 11: Table S8). The total RNA isolated from the rumen content samples was pooled and reverse-transcribed into first-strand cDNA using a ReverTra Ace Kit (Toyobo Co., Osaka, Japan). The cDNA was then subjected to PCR using the I-5™ 2 × High-Fidelity Master Mix (TsingKe Biotech, Beijing, China). The PCR cycling conditions included an initial denaturation step (98 °C for 5 min), 15 cycles of amplification (98 °C for 10 s; 52 °C for 15 s; 72 °C for 45 s) followed by another 25 cycles of amplification (98 °C for 10 s; 60 °C for 15 s; 72 °C for 45 s), and a final extension step (72 °C for 5 min). The PCR products were stored at 4 °C if not subjected to size verification on agarose (1.0%, w/v) gels. If the expected size were obtained, the product was immediately cloned into the pET-28a (+) vector by homologous recombination using the Trelief™ SoSoo Cloning Kit (TsingKe Biotech, Beijing, China). Then, the ligation products were each transformed into competent *E. coli* DH5α (TsingKe Biotech, Beijing, China) aided by heat shock. Transformants were incubated in SOC medium (1 h at 37 °C) to allow expression of the antibiotic resistance gene and then plated onto LB agar plates contains kanamycin (50 µg ml^−1^) and incubated overnight at 37 °C. Positive clones were screened using colony PCR using the T7 promoter and T7 terminator primers as described in the manufacturer’s protocol and subjected to Sanger sequencing to confirm the sequences. The obtained sequencing reads were compared to the predicted sequences using multiple sequence alignment with ClustalW (https://www.genome.jp/tools-bin/clustalw).

### Cloning for candidate cellulase genes

Thirty of the full-length ORFs were used for heterologous expression in *E. coli* BL21 (DE3) (Tiangen, Beijing, China) and subsequent cellulase activity assays. Briefly, the above recombinant plasmid containing each of the genes was obtained using a SanPrep Column Plasmid Mini-Preps Kit (Sangon, Shanghai, China) and was transformed into *E. coli* BL21 (DE3) by heat shock. Transformants were grown in liquid SOC (1 h at 37 °C), plated onto kanamycin (50 µg ml^−1^)-containing LB agar plates and grown overnight at 37 °C. The colonies confirmed to harbour recombinant plasmid using PCR as described above and stored in liquid cultures [LB, 20% glycerol, kanamycin (50 µg ml^−1^)] at − 80 °C.

### Assaying of substrate specificity

Positive *E. coli* transformants were cultured in 5 ml of LB medium containing 50 µg ml^−1^ kanamycin on a rotary shaker (200–220 rpm) at 37 °C overnight. One negative control clone that contained the empty pET-28a (+) vector was also included. This LB ‘seed’ cultures were then each inoculated into 50 ml of LB medium containing 50 µg ml^−1^ kanamycin and incubated with shaking at 200–220 rpm at 37 °C until OD_600_ reached 0.5. The recombinant protein was induced with 1 mM of isopropyl-β-thiogalactopyranoside (IPTG) at 25 °C for 6 h with shaking at 150 rpm. The recombinant *E. coli* cells were harvested by centrifugation at 12,000 rpm for 15 min at 4 °C and washed twice with sodium phosphate buffer (pH 7.4). The cell pellets were resuspended in 20 ml of sodium phosphate buffer (pH 7.4) and disrupted by sonication on ice, followed by centrifugation at 12,000 rpm for 15 min at 4 °C to remove the cell debris. To identify the substrate specificity of the 30 clones, the supernatant (crude extract) of each clone was evaluated for hydrolytic activity against CMC, pNPC and pNPG. Endoglucanase activity (CMCase activity) was assaying using the 3,5-dinitrosalicylic acid (DNS) method [46]. Briefly, each crude extract (250 µl) was mixed with an equal volume of 1% (w/v) CMC in 50 mM McIlvaine’s buffer (pH 6.0) and incubated at 39 °C for 10 min; then 500 µl of DNS was added and boiled at 100 °C for 15 min to terminate the reaction and develop the colour. The absorption of the reaction mixture was measured at 540 nm on a SpectraMax M5 (Molecular Devices). The exoglucanase and β-glucosidase activities were assayed using pNPC [47] and pNPG [48] (Aladdin, Shanghai, China) as substrates, respectively. The reaction mixture, which consisted of 250 μl of each crude extract and 250 μl of 2 mM pNPC or pNPG in 50 mM McIlvaine’s buffer (pH 6.0), was incubated at 39 °C for 10 min. The released *p*-nitrophenol (pNP) was measured as absorbance at 405 nm after adding 1.0 ml of 1 M Na_2_CO_3_. The crude extract was considered to have the corresponding activity if the measured average amount of hydrolytic product exceeded the activity of the negative control plus one standard deviation by at least 50% [15].

### Purification of recombinant protein and assaying of enzyme activity

The enzyme activities of the expressed proteins that exhibited activity against at least one type of cellulase substrate were further measured. Since no β-glucosidase activity was found in the substrate specificity test, glucosidase activity was not determined. To obtain sufficient amounts of protein, positive clones cultured overnight were inoculated into 200 ml of LB medium containing 50 µg ml^−1^ kanamycin. Cell pellets were obtained after 6 h of induction with IPTG and resuspended in 30 ml of sodium phosphate buffer (pH 7.4). Following sonication and centrifugation, the supernatant was then applied onto a 6 × His-tagged Ni-NTA agarose (Qiagen, Hilden, Germany) affinity chromatography column. The target proteins were eluted with 250 mM imidazole and 300 mM NaCl in phosphate buffer (pH 8.0). The protein concentration was determined using the Bradford method [49]. For endoglucanase activity, 50 μl of each purified protein solution was mixed with 450 μl of 1.0% (w/v) pre-warmed CMC in 50 mM McIlvaine’s buffer (pH 6.0) and incubated at 39 °C for 10 min, and the enzymatic hydrolytic reactions were terminated by adding 500 μl of DNS reagent. After boiling at 100 °C for 15 min, the concentration of reducing sugar was measured as absorbance at 540 nm with a series of known glucose concentrations serving as the standard. One unit of enzyme activity was defined as the amount of enzyme that produced 1 μmol of reducing sugars per min. For the assay of exoglucanase activity, the reaction mixture contained 250 μl of appropriately diluted purified protein and 250 μl of 2 mM pre-warmed pNPC in 50 mM McIlvaine’s buffer (pH 6.0) and incubated at 39 °C for 10 min. The reaction was terminated by the addition of 1.0 ml of 1.0 M Na_2_CO_3_, and then the pNP released was determined as absorbance at 405 nm with a series of known pNP concentrations being the standard. One unit of exoglucanase activity was defined as the amount of enzyme catalysing the release of 1 μmol of pNP per min.

### Effect of temperature and pH on the enzyme activity of two Cel5A cellulases

The optimum temperature for the hydrolytic activity of two expressed cellulases, Cel5A-h11 and Cel5A-h28, was evaluated by incubating the enzymes with pNPC and CMC, respectively, at temperatures ranging from 30 to 70 °C (5 °C increments) for 10 min in McIlvaine’s buffer (pH 6.0). Thermostability assay was performed by pre-incubating each cellulase at 40, 50 or 60 °C for different duration (2, 5, 10, 20, 30, 40, 50 and 60 min), followed by assaying the residual cellulase activity.

The optimum pH was estimated by incubating each cellulase with pNPC and CMC, respectively, at pH ranging from 3.0 to 10.0 in McIlvaine’s buffer (50 mM, pH 3.0–8.0), Tris–HCl buffer (0.2 M, pH 8.0–9.0) and glycine–NaOH buffer (0.2 M, pH 9.0–10.0). The assay mixtures were incubated at the optimum temperature of each cellulase for 10 min. For pH stability, each enzyme was pre-incubated at different pH (3.0–10.0) at 4 °C for 16 h, followed by measuring the remaining enzyme activity. All reactions were carried out in duplicate, and the enzyme activity was measured as described above.

## Additional files

**Additional file 1: Table S1.** Statistics of the metatranscriptomic sequencing data.

**Additional file 2: Table S2.** Summary of transcriptome assemblies.

**Additional file 3: Figure S1.** Length distribution of assembled transcripts (a) and unigenes (b), and the number of assembled transcripts and unigenes in each length interval (c). All the transcripts with similarity > 95% were clustered into one class with CD-HIT-EST. The unigene is the longest transcript of each class.

**Additional file 4: Table S3.** The number of cleaned reads mapped to the assembled unigenes.

**Additional file 5: Figure S2.** The FPKM density distribution of all transcriptomes.

**Additional file 6: Table S4.** Best hits of the identified cellulases when compared to the CAZy and NCBI nr databases.

**Additional file 7: Table S5.** Taxonomic annotation of the unigenes encoding CAZymes.

**Additional file 8: Table S6.** Numbers of unigenes and the open reading frames assigned to the selected cellulase-containing GH families.

**Additional file 9: Table S7.** The FPKM value of the putative ORFs assigned to the GH5 family.

**Additional file 10.** The amino acid sequence of the candidates assigned to the GH5 family.

**Additional file 11: Table S8.** The primers used in RT-PCR amplification of the selected ORFs assigned to the GH5 family.

## Data Availability

All data generated or analysed during this study are included in this published article and its additional files.

## Abbreviations

CAZyme: carbohydrate-active enzyme
GHs: glycoside hydrolases
ORF: open reading frame
CBMs: carbohydrate-binding modules
CMC: carboxymethylcellulose
pNPC: *p*-nitrophenyl-β-d-cellobioside
pNPG: *p*-nitrophenyl-β-d-glucopyranoside
FPKM: fragments per kilobase of transcript sequence per millions base pairs sequenced
GTs: glycosyltransferases
CEs: carbohydrate esterases
PLs: polysaccharides lyase
AAs: auxiliary activities
RSEM: RNA-Seq by expectation maximization
SRA: sequence reads archive
LCA: lowest common ancestor
BCR: blast coverage ratio
CDs: catalytic domains
IPTG: isopropyl-β-thiogalactopyranoside
DNS: 3,5-dinitrosalicylic acid
pNP: *p*-nitrophenol
