# Supplementary material for: Metatranscriptomics of the Hu sheep rumen microbiome reveals novel cellulases
Source: Biotechnol Biofuels. 2019 Jun 20;12:153. doi: 10.1186/s13068-019-1498-4 (PMC6587244; doi:10.1186/s13068-019-1498-4)
Supplement: Supplementary file 1 — Additional file 1: Table S1. Statistics of the metatranscriptomic sequencing data. [file 13068_2019_1498_MOESM1_ESM.docx]

| **Sample name** | **Raw reads** | **Clean reads** | **Clean bases (Gb)** | **Error^a^ (%)** | **Q20 (%)^b^** | **Q30 (%)^c^** | **GC (%)^d^** |
| --- | --- | --- | --- | --- | --- | --- | --- |
| S1 | 54,344,348 | 50,298,732 | 7.54 | 0.03 | 93.72 | 85.70 | 47.21 |
| S2 | 45,990,442 | 43,210,364 | 6.48 | 0.03 | 94.18 | 86.53 | 46.86 |
| S3 | 55,488,734 | 54,097,536 | 8.11 | 0.01 | 98.00 | 94.59 | 47.15 |
| S4 | 50,965,554 | 49,700,242 | 7.40 | 0.01 | 98.07 | 94.76 | 46.38 |
| S5 | 43,364,984 | 42,053,956 | 6.31 | 0.02 | 97.42 | 93.09 | 49.19 |
| S6 | 43,902,358 | 43,089,054 | 6.46 | 0.01 | 97.66 | 93.65 | 46.43 |

^a^ Base error rate

^b, c^ The percentage of bases with Phred values greater than 20 (b) and 30 (c) as a percentage of total bases

^d^ The base G+C %
