# Supplementary material for: Metatranscriptomics of the Hu sheep rumen microbiome reveals novel cellulases
Source: Biotechnol Biofuels. 2019 Jun 20;12:153. doi: 10.1186/s13068-019-1498-4 (PMC6587244; doi:10.1186/s13068-019-1498-4)
Supplement: Supplementary file 2 — Additional file 2: Table S2. Summary of transcriptome assemblies. [file 13068_2019_1498_MOESM2_ESM.docx]

| **Assembly features** | **Statistics** |
| --- | --- |
| Numbers of unigenes | 2,380,783 |
| Number of long unigene (>=1000 bp) | 170,628 |
| Max unigene length (bp) | 40,135 |
| Mean unigene length (bp) | 515 |
| N50 (bp) | 530 |
