# Supplementary figures and images for: Metatranscriptomics of the Hu sheep rumen microbiome reveals novel cellulases
Source: Biotechnol Biofuels. 2019 Jun 20;12:153. doi: 10.1186/s13068-019-1498-4 (PMC6587244; doi:10.1186/s13068-019-1498-4)

**a**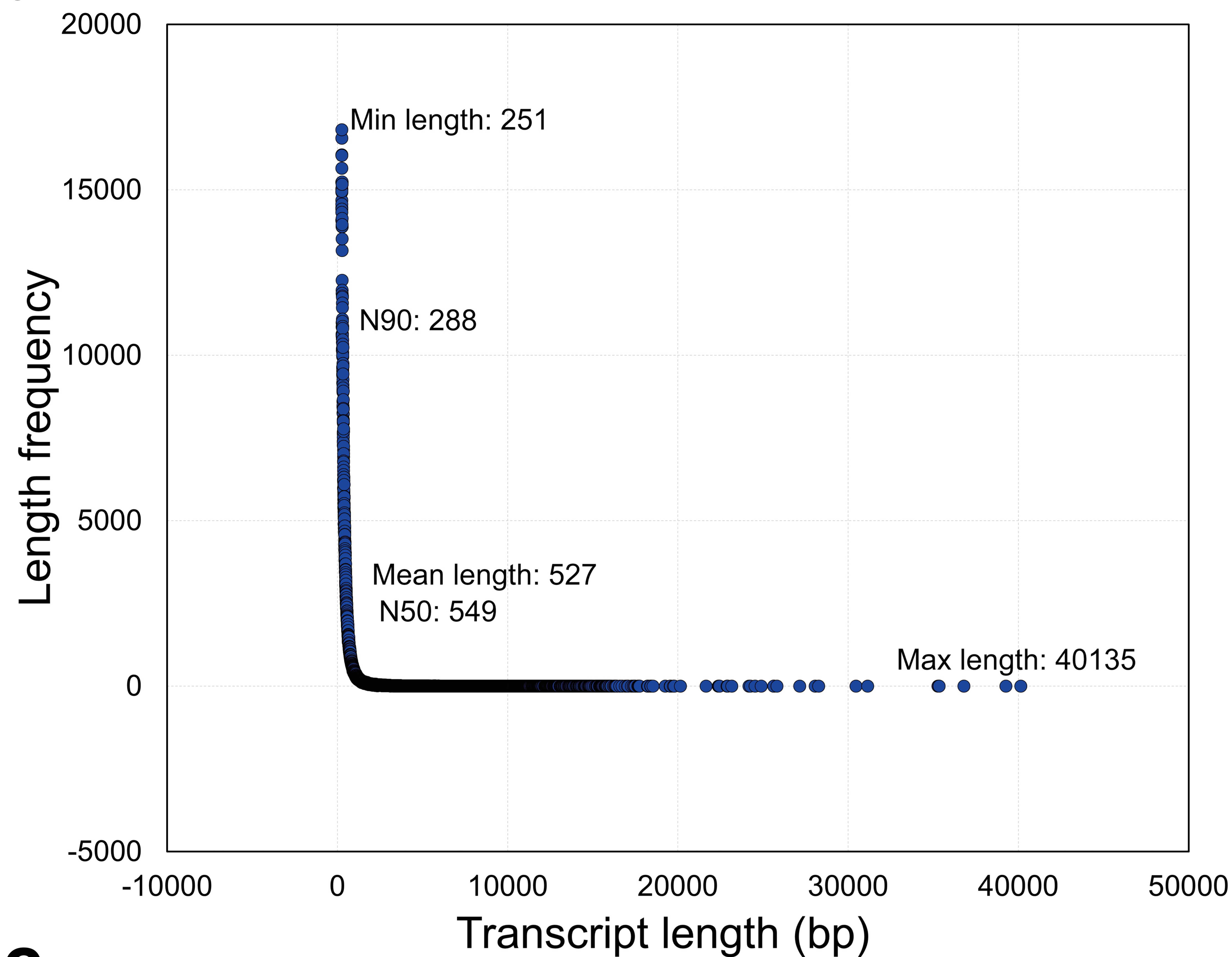**b**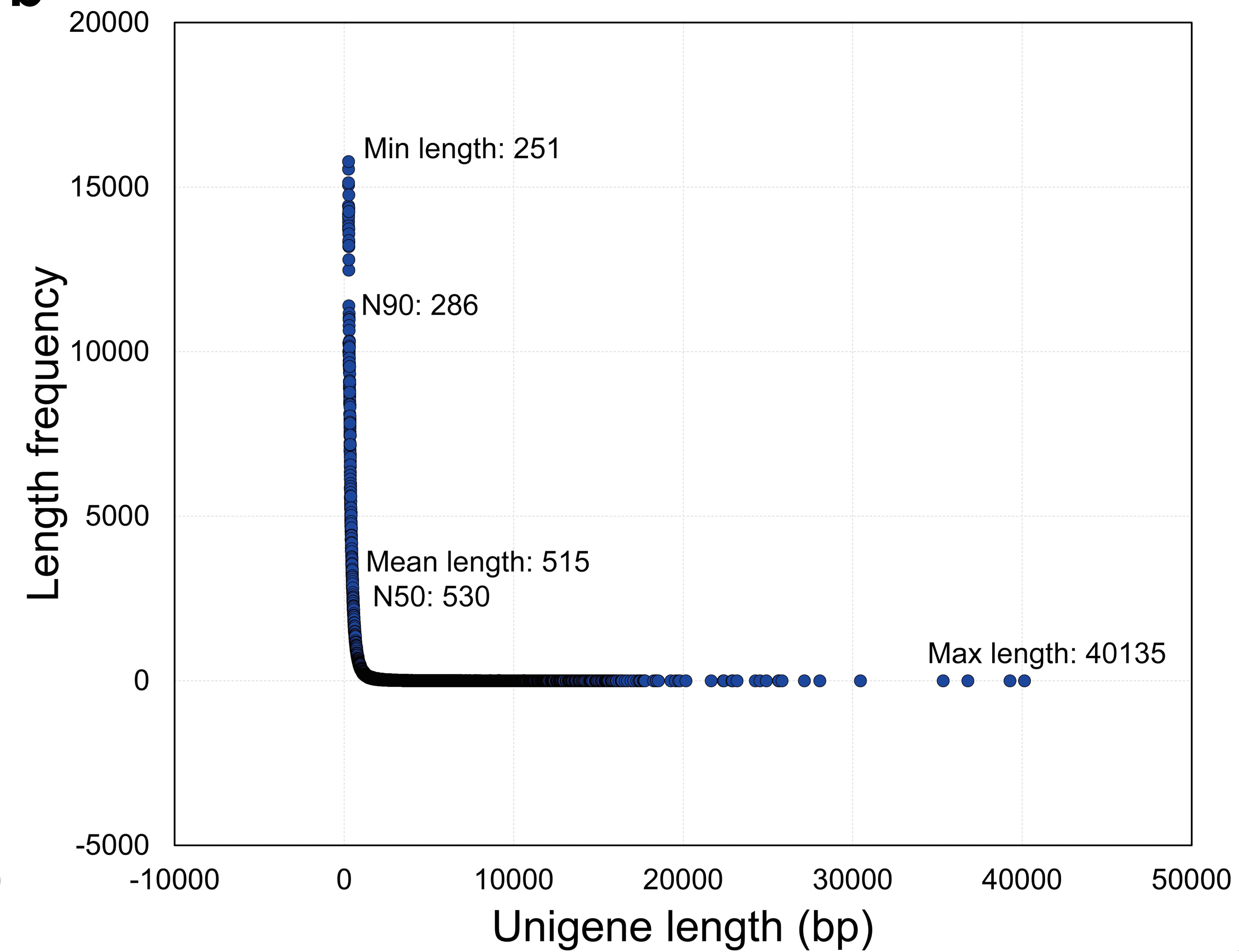**c**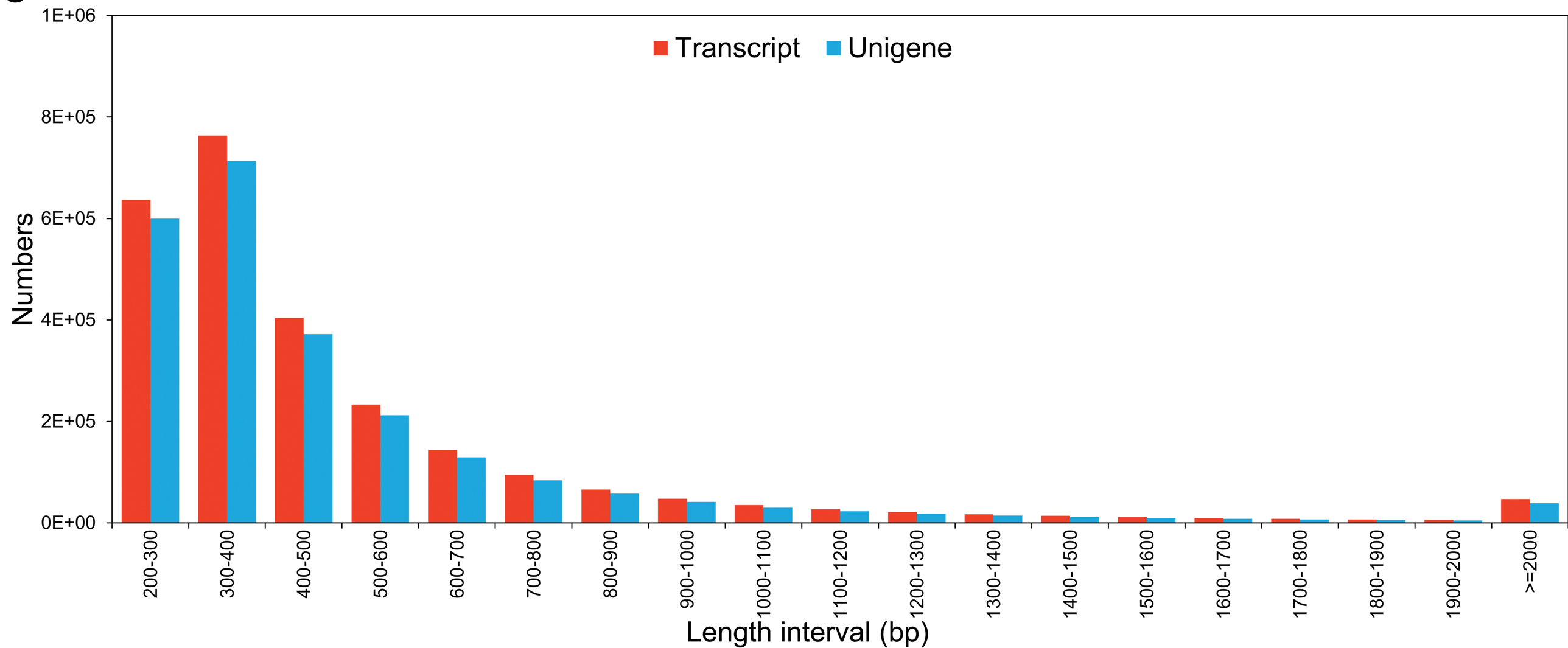

Supplement: Supplementary file 3 — Additional file 3: Figure S1. Length distribution of assembled transcripts (a) and unigenes (b), and the number of assembled transcripts and unigenes in each length interval (c). All the transcripts with similarity > 95% were clustered into one class with CD-HIT-EST. The unigene is the longest transcript of each class. [file 13068_2019_1498_MOESM3_ESM.pdf]

FPKM density distribution

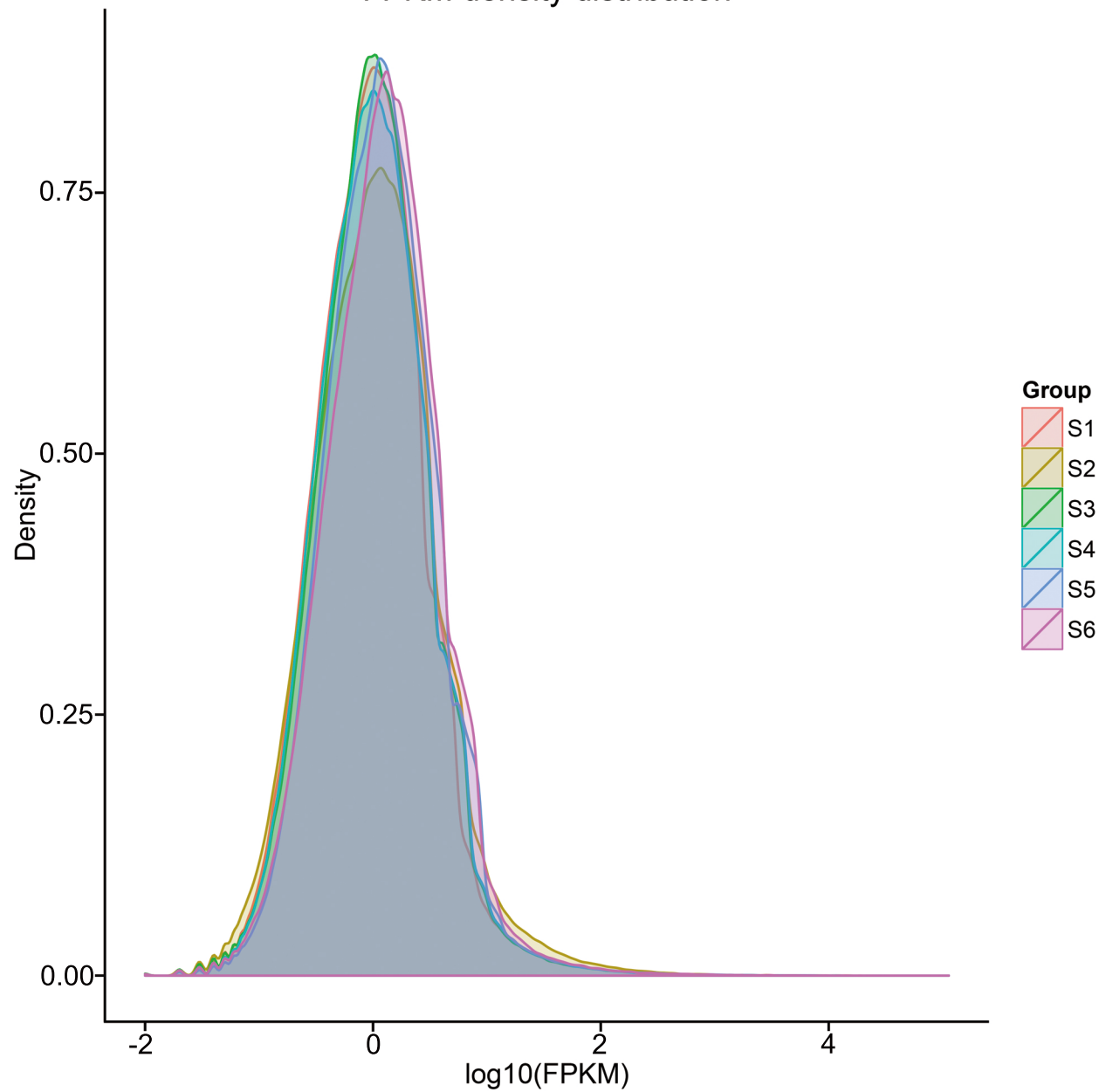

Supplement: Supplementary file 5 — Additional file 5: Figure S2. The FPKM density distribution of all transcriptomes. [file 13068_2019_1498_MOESM5_ESM.pdf]
