# Supplementary material for: Metatranscriptomics of the Hu sheep rumen microbiome reveals novel cellulases
Source: Biotechnol Biofuels. 2019 Jun 20;12:153. doi: 10.1186/s13068-019-1498-4 (PMC6587244; doi:10.1186/s13068-019-1498-4)
Supplement: Supplementary file 4 — Additional file 4: Table S3. The number of cleaned reads mapped to the assembled unigenes. [file 13068_2019_1498_MOESM4_ESM.docx]

| **Sample name** | **Total reads** | **Total mapped** |
| --- | --- | --- |
| S1 | 50,298,732 | 23,939,218 (47.6%) |
| S2 | 43,210,364 | 20,629,686 (47.7%) |
| S3 | 54,097,536 | 25,513,336 (47.2%) |
| S4 | 49,700,242 | 24,017,720 (48.3%) |
| S5 | 42,053,956 | 18,754,386 (44.6%) |
| S6 | 43,089,054 | 20,810,156 (48.3%) |
