# Supplementary material for: Metatranscriptomics of the Hu sheep rumen microbiome reveals novel cellulases
Source: Biotechnol Biofuels. 2019 Jun 20;12:153. doi: 10.1186/s13068-019-1498-4 (PMC6587244; doi:10.1186/s13068-019-1498-4)
Supplement: Supplementary file 8 — Additional file 8: Table S6. Numbers of unigenes and the open reading frames assigned to the selected cellulase-containing GH families. [file 13068_2019_1498_MOESM8_ESM.docx]

| **GH family** | **Number of unigenes** | **Number of open reading frames (ORFs)** |
| --- | --- | --- |
| GH1 | 1,142 | 277 |
| GH3 | 6,242 | 1,795 |
| GH5 | 2,887 | 870 |
| GH6 | 38 | 5 |
| GH8 | 291 | 111 |
| GH9 | 1,681 | 567 |
| GH12 | 17 | 0 |
| GH30 | 542 | 157 |
| GH44 | 121 | 23 |
| GH45 | 98 | 19 |
| GH48 | 120 | 33 |
| GH51 | 1,032 | 282 |
| GH74 | 202 | 55 |
| GH116 | 31 | 12 |
| GH124 | 45 | 19 |
| Total | 14,489 | 4,225 |
